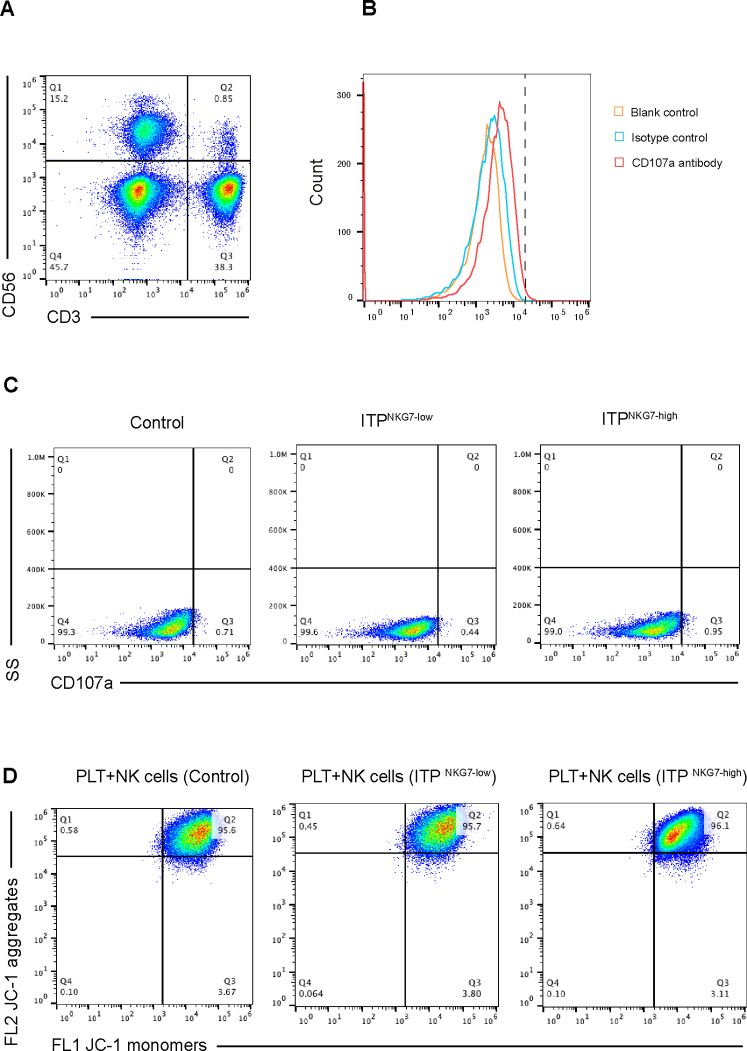# Natural killer cell granule protein 7 contributes to CD8^+^ T cell-mediated platelet apoptosis in immune thrombocytopenia

**DOI:** 10.1016/j.rpth.2025.102977

**Published:** 2025-07-22

**Authors:** Xiaolin Wang, Qiang Liu, Xiaojing Li, Wei Xing, Ping Chen, Qi Feng, Ming Hou, Qian Wang, Hai Zhou, Jun Peng

**Affiliations:** 1 Department of Hematology, Qilu Hospital of Shandong University, Jinan, China; 2 Department of Clinical Laboratory, Qilu Hospital (Qingdao) of Shandong University, Qingdao, China; 3 Fujian Institute of Hematology, Fujian Provincial Key Laboratory on Hematology, Fujian Medical University Union Hospital, Fuzhou, China; 4 Department of Geratology, The Second Affiliated Hospital of Shandong University of Traditional Chinese Medicine, Jinan, China; 5 Department of Clinical Laboratory, Qilu Hospital of Shandong University, Jinan, China

**Keywords:** blood platelets, CD8-positive T lymphocytes, cytotoxicity immunologic, NKG7 protein, human, purpura, thrombocytopenic, idiopathic

## Abstract

**Background:**

CD8^+^ T cells participate in the pathogenesis of primary immune thrombocytopenia (ITP). Natural killer cell granule protein 7 (NKG7) is essential for natural killer cell and CD8^+^ T cell cytotoxicity. The function of NKG7 in CD8^+^ T cells in ITP remains unclear.

**Objectives:**

We investigated the expression and roles of NKG7 in CD8^+^ T cells in ITP.

**Methods:**

We analyzed NKG7 and CD107a expression and CD8^+^ T cell-mediated platelet apoptosis in patients with ITP and controls. NKG7 knockdown was performed using small interfering RNA, and the extracellular signal-regulated kinases 1 and 2 pathway was analyzed by western blot analysis.

**Results:**

NKG7 was significantly increased in CD8^+^ T cells and positively correlated with CD107a and CD8^+^ T cell-induced platelet apoptosis in ITP. Based on NKG7 levels, patients with ITP were divided into NKG7 high-expression and low-expression groups. Patients with high expression of NKG7 had significantly higher levels of CD107a and CD8^+^ T cell-induced platelet apoptosis than controls, whereas no difference was found between patients with low NKG7 expression and controls. Platelet counts in patients with high NKG7 expression were significantly lower than those in patients with low NKG7 expression. We knocked down NKG7 in CD8^+^ T cells from patients with ITP and found decreased CD107a expression and less platelet apoptosis *in vitro*. We further found that NKG7 affected the cytotoxicity of CD8^+^ T cells through the extracellular signal-regulated kinase 1 and 2 pathway.

**Conclusion:**

NKG7 plays an important role in CD8^+^ T cell-mediated cytotoxicity might be a potential therapeutic target for ITP.

## Essentials

• Natural killer cell granule protein 7 (NKG7) is essential for CD8^+^ T cell-mediated cytotoxicity.
• We analyzed the effect of NKG7 in CD8^+^ T cells in primary immune thrombocytopenia (ITP).
• NKG7 was increased and positively correlated with CD8^+^ T cell-induced cytotoxicity in ITP.
• NKG7 can be used as an important indicator of cytotoxicity and as a therapeutic target for ITP.

## Graphical abstract

**Figure:**
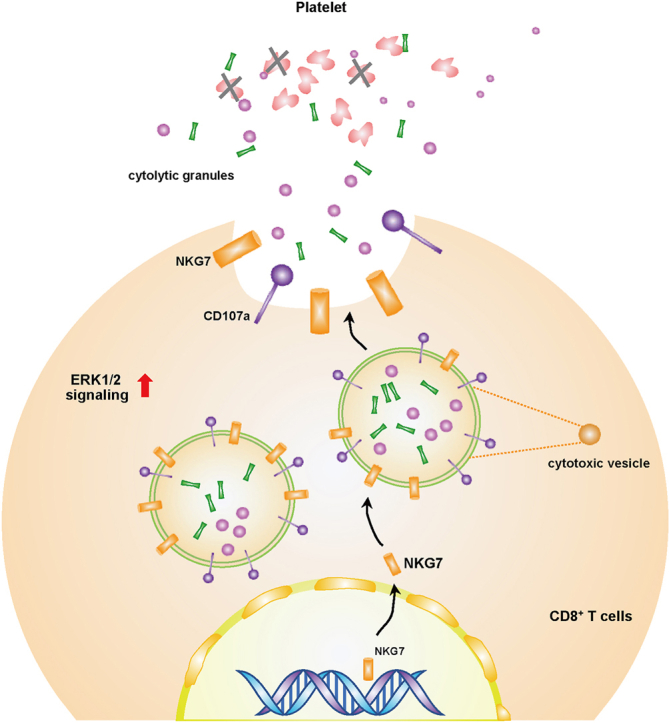

## 1 Introduction

Primary immune thrombocytopenia (ITP) is an acquired autoimmune disorder characterized by isolated thrombocytopenia due to accelerated platelet destruction and impaired platelet production [[1], [2], [3], [4]]. The incidence of ITP in adults ranges from 1.6 to 3.9 per 100,000 person-years [5]. Patients predominantly present with varying degrees of bleeding, from mild mucocutaneous to life-threatening bleeding. Approximately 15% of patients with ITP require hospitalization due to bleeding, and intracranial hemorrhage is the most severe bleeding complication [6,7]. ITP is traditionally considered to be an autoantibody-mediated disorder [8,9]; however, platelet autoantibodies can only be detected in 50% to 70% of patients [6,10,11]. In addition to platelet autoantibodies, CD8^+^ T cell-mediated platelet lysis plays an important role in the pathogenesis of ITP [[12], [13], [14], [15], [16], [17]]. Previous studies found that the percentage of CD8^+^ T cells was higher and CD8^+^ T cell-mediated platelet apoptosis was increased in patients with active chronic ITP than in normal controls and remissive patients [12,18,19].

The granule-dependent pathway is an important mechanism by which CD8^+^ T cells mediate targeted cell death. Cytotoxic granules such as perforin and granzyme B are released from lysosomes into the immunologic synapse, ultimately resulting in activation of the cell death pathway [20,21]. During this period, lysosome-associated membrane protein-1 (LAMP-1, also known as CD107a), an important protein located on the luminal sides of cytotoxic granules, is expressed on plasma membrane of CD8^+^ T cells after the release of granule contents [[21], [22], [23]]. Previous reports have verified that the expression of CD107a is associated with degranulation and directly correlates with cytotoxic activity of CD8^+^ T cells. In other words, CD107a is a sensitive marker for cytotoxic activity [[22], [23], [24], [25]]. In this study, we also used CD107a and CD8^+^ T cell-induced platelet apoptosis as a measure of cytotoxicity of CD8^+^ T cells in patients with ITP and controls.

Natural killer cell granule protein 7 (NKG7), also known as GMP-17 [26] or GIG-1 [27], is an integral membrane protein that was first found to be predominantly expressed in T cells and natural killer (NK) cells [28]. NKG7 can localize to vesicles containing cytotoxic granules and translocates to the cell surface after target cell recognition [26,29]. Recent studies have reported that NKG7 is a regulator of granule exocytosis of CD8^+^ T cells in a variety of diseases, such as inflammatory and autoimmune disorders and cancer [[30], [31], [32], [33]]. CD8^+^ T cells lacking NKG7 have a reduced capacity to degranulate. The depletion of NKG7 by small interfering RNA (siRNA) resulted in altered number and size of cytolytic granules, as well as their abnormal trafficking and release [31,32]. Furthermore, NKG7 can enhance CD8^+^ T cell immune synapse efficiency and reduce the production of potentially harmful inflammatory factors, including IL-7, IL-12, IL-10, IL-2, TNF-α, and IL-13 [34]. Additionally, NKG7 plays a critical role in antitumor immunotherapy. Studies have confirmed that NKG7 can increase the accumulation and activation of T cells in the tumor microenvironment [35]. NKG7 in CD8^+^ T cells is also elevated in some cancer patients with durable response to immunotherapy [33]. However, the effect of NKG7 in CD8^+^ T cell-mediated platelet destruction in ITP remains unclear.

Herein, we found that NKG7 has a vital effect on CD8^+^ T cell-mediated platelet apoptosis in ITP. First, the expression of NKG7 in CD8^+^ T cells was higher in patients with ITP than in healthy controls. Second, NKG7 was positively correlated with CD107a and CD8^+^ T cell-associated platelet apoptosis in patients with ITP. Third, we found that compared with patients with low NKG7 expression, patients with high NKG7 expression had significantly higher cytotoxic activity of CD8^+^ T cells and lower peripheral platelet counts. We knocked down NKG7 in CD8^+^ T cells using siRNA and found that CD8^+^ T cells exhibited decreased cytotoxicity. Knockdown of NKG7 reduced the phosphorylation of extracellular signal-regulated kinases 1 and 2 (ERK1/2) and decreased the expression of LAMP1. Therefore, we demonstrated that NKG7 plays an important role in CD8^+^ T cell-mediated cytotoxicity in ITP.

## 2 Methods

### 2.1 Patients and controls

Forty-three patients newly diagnosed with ITP (23 women and 20 men; age range 18-81 years, median 47 years) were recruited in the Department of Hematology, Qilu Hospital, Shandong University between January 2023 and December 2024. All patients fulfilled the clinical diagnostic criteria for ITP as previously published [1]. The disease duration in these patients was <3 months, and they had not received any treatment. Secondary ITP, which primarily includes autoimmune diseases and medication- or tumor-induced thrombocytopenia, was excluded after a review of the patients’ medical history, physical examination, and laboratory tests. Bleeding scores were calculated at the time of admission according to bleeding rating system for ITP [36,37]. This system includes 2 indicators: age and bleeding manifestation. The bleeding score is the sum of the age score and bleeding symptom score (the highest score among all bleeding scores). This bleeding rating system is very consistent with ITP-specific bleeding assessment tool (ITP-BAT) [38] and information collection requires less time [39]. The control group consisted of 23 healthy volunteers (12 women and 11 men; age range 17-69 years, median 40 years). Platelet counts and baseline characteristics were recorded at the time of sample collection. All studies were approved by the Medical Ethical Committee of Qilu Hospital, Shandong University, and informed consent was obtained from all participants in accordance with the Declaration of Helsinki.

### 2.2 Cell separation and stimulation

Whole blood from patients with ITP and healthy donors was drawn from peripheral venous blood vessels into tubes containing ethylenediaminetetraacetic acid. Venous blood was centrifuged (300*g*, 8 minutes, 22 °C) immediately to obtain the platelet-rich plasma and further centrifuged (1250*g*, 10 minutes, 22 °C) to separate platelets from the platelet-rich plasma. Platelets were resuspended in RPMI 1640 medium supplemented with 10% heat-inactivated fetal bovine serum (Gibco) and adjusted to 10^6^/mL prior to use. Peripheral blood mononuclear cells (PBMCs) were isolated using Ficoll–Hypaque density gradient centrifugation (Cytiva). PBMCs were cultured in RPMI 1640 medium supplemented with 10% heat-inactivated fetal bovine serum and were stimulated with phorbol myristate acetate (50 ng/mL, Sigma-Aldrich) and ionomycin (50 ng/mL, Sigma-Aldrich) at 37 °C in a humidified atmosphere containing 5% CO_2_ for 12 hours. The cells were then harvested for further experiments.

### 2.3 *Ex vivo* culture system

CD8^+^ T cells were enriched from PBMCs using CD8 magnetic microbeads and mass spectrometry columns (Miltenyi Biotec). Isolated CD8^+^ T cells were stained with APC-conjugated anti–human CD8 (BioLegend) for 30 minutes, then the proportion of CD8^+^ T cells was analyzed by flow cytometry. The purity of enriched CD8^+^ T cells was >90%. CD8^+^ T cells isolated from patients and healthy donors were used as effector cells, and autologous platelets were used as target cells. CD8^+^ T cells were incubated with platelets at a ratio of 1:10 in complete media (5% CO_2_, 37 °C). After 4 hours, cells were collected to analyze CD8^+^ T cell-induced platelet apoptosis. NK cells were also enriched from PBMCs using a NK cell isolation kit and mass spectrometry columns (Miltenyi Biotec). The purity of enriched NK cells was ∼88%. The protocol for incubation with platelets was consistent with that used for CD8^+^ T cells. After 4 hours, platelets were collected to analyze NK cell-induced platelet apoptosis.

### 2.4 Flow cytometry analysis

**Supplementary FigS1:**
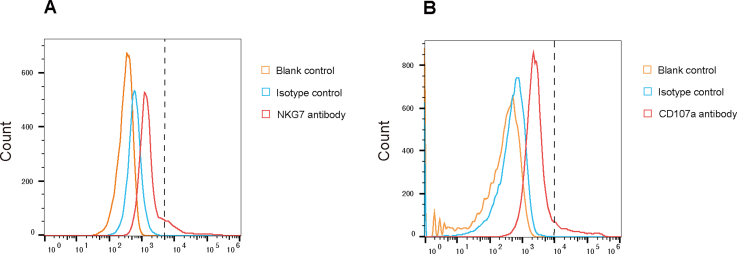


PBMCs were stained with APC-conjugated anti–human CD8 (BioLegend) for 30 minutes, rabbit anti–human NKG7 (E6S2A) monoclonal antibody (mAb; Cell Signaling Technology) for 1 hour, and Alexa Fluor488–conjugated anti–rabbit IgG (H+L) (Cell Signaling Technology) for 30 minutes. The expression of NKG7 was analyzed. Cells were also stained with PE/Cyanine7-conjugated anti–human CD107a (BioLegend) for 30 minutes to assess the expression of CD107a. Rabbit (DA1E) mAb IgG XP Isotype Control (Cell Signaling Technology) was used as the isotype control antibody for anti–human NKG7 (E6S2A) Rabbit mAb. PE/Cyanine7 Mouse IgG1, κ Isotype Ctrl Antibody (BioLegend) was used as the isotype control antibody for PE/Cyanine7-conjugated anti–human CD107a. We established the gates for NKG7^+^ CD8^+^ and CD107a^+^ CD8^+^ T cells based on these isotype-matched control antibodies (Supplementary Figure 1A, B). We also investigated NKG7 expression on NK cells from patients with ITP and healthy controls. Cells were stained with PE-conjugated anti–human CD3 (BioLegend), PE/Cyanine7-conjugated anti–human CD16 (BioLegend), and APC-conjugated anti–human CD56 (BioLegend) for 30 minutes to identify NK cells, and then cells were stained for NKG7 according to the above method. Additionally, we analyzed CD107a expression on NK cells. Cells were stained with PE-conjugated anti–human CD3 (BioLegend), APC-conjugated anti–human CD56 (Biolegend), and PE/Cyanine7-conjugated anti–human CD107a (BioLegend) for 30 minutes to analyze CD107a expression on NK cells by flow cytometry.

CD8^+^ T cells and platelets were cultured in complete medium for 4 hours to assess platelet apoptosis. Platelets were harvested and incubated with APC-conjugated anti–human CD61 (Biolegend). Then, platelets were stained with JC-1 (5,5′,6,6′-tetrachloro-1,1′,3,3′-tetraethylbenzimidazolylcarbocyanine iodide) using a mitochondrial membrane potential assay kit (Beyotime). Mitochondrial transmembrane potential (ΔΨ_m_) depolarization is a hallmark event in the early stage of platelet apoptosis. The ΔΨ_m_ of platelets is evaluated using the fluorescent probe JC-1, which is a monomer molecule permeable to the mitochondrial membrane [40,41]. If ΔΨ_m_ is maintained, JC-1 accumulates in the mitochondria, resulting in a fluorescence shift from green (monomeric form) to red (aggregate form). However, if ΔΨ_m_ is depolarized, JC-1 fails to accumulate in the mitochondria and the fluorescence remains green. Therefore, platelets with depolarized ΔΨ_m_ can be evaluated by quantifying the fraction of cells that exclusively exhibit green fluorescence. CD8^+^ T cell-induced platelet apoptosis was calculated by subtracting spontaneous apoptosis from total apoptosis in cocultures. The detection method for platelet apoptosis mediated by NK cells was the same as above. All cells were performed using a Gallios Flow Cytometer (Beckman Coulter), and FlowJo software (Treestar) was used for analysis.

### 2.5 Real-time quantitative PCR (RT-qPCR)

Total RNA was extracted using the RNA-Quick Purification Kit (YiShan Biotechnology) according to the manufacturer’s instructions. The concentration of RNA (ng/μL) and sample purity (260/280 ratio) was measured using a NanoDrop 2000 UV-Vis Spectrophotometer (Thermo Fisher Scientific). Extracted RNA was converted into cDNA using the Evo M-MLV RT Kit (Accurate Biology) according to the manufacturer’s instructions. mRNA expression of *NKG7* and glyceraldehyde-3-phosphate dehydrogenase (*GAPDH*; endogenous control) in CD8^+^ T cells was quantified by RT-qPCR on a LightCycler 480 System (Roche Applied Science). After hot start at 95 °C for 10 minutes, PCR was performed as follows: denaturation at 95 °C for 15 seconds, annealing at 60 °C for 15 seconds, and extension at 72 °C for 45 seconds for a total of 45 cycles. Relative of changes in gene expression were calculated using the 2^−ΔΔCt^ method. The primer sequences used were as follows: NKG7 (forward): 5′-TTCTACCTGGGCTGGGTCTC-3′, NKG7 (reverse): 5′-GGTTTCATAGCCAGGACGGG-3′; GAPDH (forward): 5′-GCACCGTCAAGGCTGAGAAC-3′, GAPDH (reverse): 5′-TGGTGAAGACGCCAGTGGA-3′.

### 2.6 Knockdown of NKG7 by siRNA

CD8^+^ T cells were isolated from patients with ITP and seeded at 3 × 10^6^ cells per well. Cells were cultured at 37 °C for 12 hours and then transferred to Opti-MEM I reduced serum medium (Gibco). CD8^+^ T cells were transfected with 60 nM of siRNA (sense GGCCUGAUGUUCUGCCUGA and antisense UCAGGCAGAACAUCAGGCC; SyngenTech) against NKG7 by using Lipofectamine RNAiMAX transfection reagent (Invitrogen). The negative control siRNA sequences were UUCUCCGAACGUGUCACGU and ACGUGACACGUUCGGAGAA. Experiments were conducted in accordance with the manufacturer’s instructions. After 6 hours, the medium was replaced with RPMI 1640 medium with 10% fetal bovine serum, anti–human CD3 (1 ng/mL), anti–human CD28 antibody (1 ng/mL), and recombinant human IL-2 (10 ng/mL). Forty-eight hours after the transfection, subsequent analyses were carried out. The expression of *NKG7* mRNA in cells transfected with siNKG7 and those with negative control siRNA was tested by quantitative PCR.

### 2.7 Western blot analysis

CD8^+^ T cells from controls and CD8^+^ T cells from patients with ITP transfected with siNKG7 were lysed with precooled RIPA lysate buffer with 1% protease inhibitor and 1% protein phosphatase inhibitor (Beyotime). The proteins in supernatants were separated by sodium dodecyl sulfate polyacrylamide gel electrophoresis and transferred onto a polyvinylidene fluoride membrane (Merck Millipore). Then, the membrane was blocked with 5% skim milk in Tris-buffered saline for 1 hour. The membranes were incubated with primary antibodies at 4 °C overnight and with the secondary antibody at room temperature for 1 hour. Finally, the protein bands were detected with ECL Reagent (Merck Millipore) in a darkroom. Primary antibodies used were as follows: anti–phospho-p44/42 MAPK (Erk1/2) (1:2000, Cell Signaling Technology), anti–p44/42 MAPK (Erk1/2) (1:1000, Cell Signaling Technology), anti-LAMP1 (1:1000, Cell Signaling Technology), anti-GAPDH (1:10000, abcam), anti–β-actin (1:1000, Cell Signaling Technology), and anti–rabbit IgG H&L (1:10,000, abcam).

### 2.8 Statistical analysis

Continuous nonparametric variables were analyzed using the Mann–Whitney U-test and are reported as median (IQR). Continuous parametric variables were analyzed using the Student’s *t*-test and are reported as mean ± SEM. Categorical data were analyzed using the chi-squared test. The correlation between 2 sets of variables was determined by Pearson’s correlation. The optimal cutoff value was identified by receiver operating characteristic curve analysis with the best specificity and sensitivity. All statistical analyses were performed using SPSS version 27.0 (SPSS Software Inc) and GraphPad Prism 9 (GraphPad Software), and a 2-tailed *P* value <.05 was considered statistically significant.

## 3 Results

### 3.1 NKG7 expression was higher in CD8^+^ T cells from patients with ITP than those from controls

**Figure 1:**
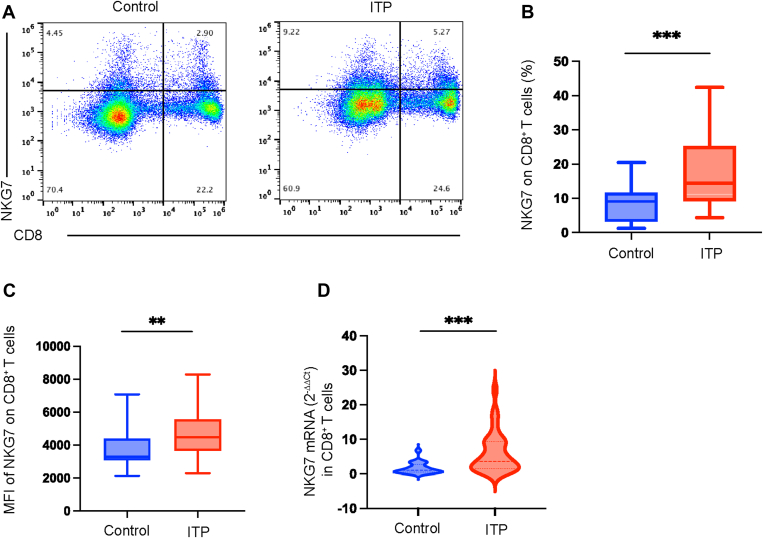
Natural killer cell granule protein 7 (NKG7) expression in CD8^+^ T cells is higher in patients with immune thrombocytopenia (ITP) than in healthy controls. (A) Flow cytometric analysis of NKG7 expression in CD8^+^ T cells in patients with ITP and controls. (B) NKG7 expression in CD8^+^ T cells in patients with ITP was significantly higher than that in controls (*P* < .001). (C) The mean fluorescence intensity (MFI) of NKG7^+^ CD8^+^ T cells was higher in patients with ITP than in controls (*P* = .006). (D) Violin plot showing that patients with ITP had markedly higher levels of *NKG7* mRNA expression than healthy controls (*P* < .001). ∗∗*P* < .01, ∗∗∗*P* < .001. ITP: *n* = 43, control: *n* = 23.

In the present study, we analyzed NKG7 expression on the surface of CD8^+^ T cells from both patients with ITP and healthy controls by flow cytometry. We found that the expression of NKG7 was higher in patients with ITP than in controls (16.90% ± 1.43% vs 9.19% ± 1.22%, patients vs controls, *P* < .001; Figure 1A, B). We also analyzed the mean fluorescence intensity (MFI) of NKG7 on CD8^+^ T cells. We found that the MFI of NKG7^+^ CD8^+^ T cells was higher in patients with ITP than in controls (4813 ± 256.4 vs 3690 ± 247.1, patients vs controls, *P* = .006; Figure 1C). Moreover, the total *NKG7* mRNA expression in CD8^+^ T cells from patients with ITP was significantly increased compared with that of healthy controls (3.66, IQR: 1.52, 9.41 vs 1.00, IQR: 0.48, 2.75, patients vs controls, *P* < 0.001; Figure 1C) (ITP: *n* = 43, control: *n* = 23).

### 3.2 NKG7 is positively correlated with CD8^+^ T cell-mediated cytotoxicity in patients with ITP

**Figure 2:**
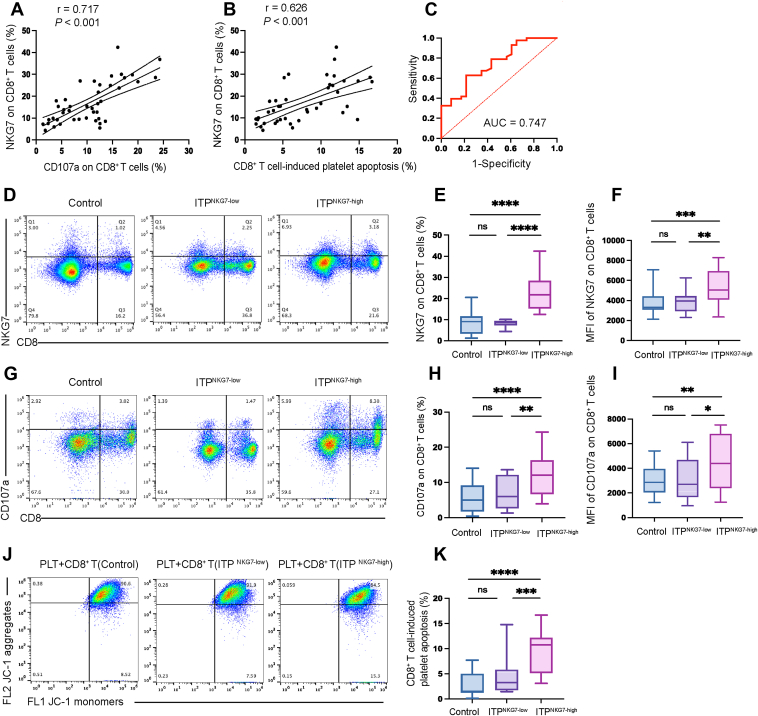
Natural killer cell granule protein 7 (NKG7) expression is positively correlated with CD8^+^ T cell-mediated cytotoxicity. (A) NKG7 expression was positively correlated with CD107a on CD8^+^ T cells (*r* = 0.717, *P* < .001); *n* = 43. (B) NKG7 expression was positively correlated with CD8^+^ T cell-induced platelet apoptosis (*r* = 0.626, *P* < .001); *n* = 43. (C) Receiver operating characteristic (ROC) curve analysis was performed to determine the optimal cutoff value with the best specificity and sensitivity. The area under the ROC curve (AUC) was 0.747 (95% CI: 0.626-0.869; *P* = .001). Patients were divided into 2 groups: NKG7 high-expression group (*n* = 27) and NKG7 low-expression group (*n* = 16). (D) Flow cytometric analysis of NKG7 expression in controls, NKG7 low-expression immune thrombocytopenia (ITP) patients, and NKG7 high-expression patients with ITP. (E) The proportion of NKG7^+^ CD8^+^ T cells was significantly higher in NKG7 high-expression patients with ITP than in both controls (*P* < .001) and NKG7 low-expression patients (*P* < .001). No difference was observed between NKG7 low-expression patients with ITP and controls (*P* = .40). (F) The mean fluorescence intensity (MFI) of NKG7 is elevated in NKG7 high-expression patients compared with controls (*P* < .001) and NKG7 low-expression patients (*P* = .002). There was no difference between NKG7 low-expression patients and controls (*P* = .70). (G) Flow cytometric analysis of CD107a expression in controls, NKG7 low-expression patients with ITP, and NKG7 high-expression patients. (H) The proportion of CD107a^+^ CD8^+^ T cells was increased in NKG7 high-expression patients compared with healthy controls (*P* < .001) and NKG7 low-expression patients (*P* = .002). There was no difference between NKG7 low-expression patients with ITP and controls (*P* = .37). (I) CD107a MFI was elevated in NKG7 high-expression patients compared with controls (*P* = .002) and NKG7 low-expression patients (*P* = .02). There was no difference between NKG7 low-expression patients and controls (*P* = .80). (J) Flow cytometric analysis of CD8^+^ T cell-induced platelet apoptosis in controls, NKG7 low-expression patients with ITP, and NKG7 high-expression patients with ITP. Platelet apoptosis was assessed by their depolarized ΔΨ_m_ using the fluorescent probe JC-1. JC-1 aggregates reflect potential-dependent accumulation in the mitochondria, whereas JC-1 monomers represent the monomeric form of JC-1 after mitochondrial ΔΨ_m_ depolarization. Platelet apoptosis can be evaluated by measuring the percentage of JC-1 monomers only. (K) The percentage of platelet apoptosis was increased in NKG7 high-expression patients compared with that in controls (*P* < .001) and NKG7 low-expression patients (*P* < .001). There was no difference between NKG7 low-expression patients and controls (*P* = .12). ∗*P* < .05, ∗∗*P* < .01, ∗∗∗*P* < .001, ∗∗∗∗*P* < .0001; ns, not significant; PLT, platelet. Control: *n* = 23, NKG7 high-expression group: *n* = 27, NKG7 low-expression group: *n* = 16.

We analyzed the relevance of NKG7 and the cytotoxicity of CD8^+^ T cells from patients with ITP using Pearson’s correlation coefficient analysis. We found that NKG7 was positively correlated with CD107a (*r* = 0.717, *P* < .001; Figure 2A). Similarly, there was a significantly positive correlation between NKG7 and CD8^+^ T cell-induced platelet apoptosis (*r* = 0.626, *P* < .001, Figure 2B) (*n* = 43).

### 3.3 Patients with high NKG7 expression had significantly higher CD8^+^ T cell-mediated cytotoxicity than controls and patients with low NKG7 expression

NKG7 expression in patients with ITP was significantly higher than that in healthy controls, as shown in Figure 1B, but some patients had relatively low expression of NKG7. To categorize patients with ITP based on NKG7 levels and explore the differences in CD8^+^ T cell cytotoxicity, we performed receiver operating characteristic curve analysis and determined that the optimal cutoff value with the maximal sensitivity and specificity was 11.99% (Figure 2C). As a result, 27 patients with NKG7 levels above the cutoff value were grouped into the high-expression group, while the remaining 16 patients were classified into the low-expression group. The high NKG7 expression subgroup constituted 62.79% of the study cohort. Patients with high NKG7 expression had significantly higher levels of NKG7 than both controls (22.13% ± 1.54% vs 9.19% ± 1.22%, high-expression group vs control, *P* < 0.001) and patients with low NKG7 expression (22.13% ± 1.54% vs 8.08% ± 0.43%, high-expression group vs low-expression group, *P* < 0.001). No difference was observed between patients with low NKG7 expression and controls (Figure 2D, E). The MFI of NKG7 was elevated in patients with high NKG7 expression, compared with that of controls (5393 ± 332.9 vs 3690 ± 247.1, high-expression group vs control, *P* < .001) and patients with low NKG7 expression (5393 ± 332.9 vs 3833 ± 262.3, high-expression group vs low-expression group, *P* = 0.002). There was no difference between patients with low NKG7 expression and controls (Figure 2F).

Patients with high NKG7 expression had significantly higher levels of CD107a than both controls (12.37% ± 1.08% vs 5.73% ± 0.85%, high-expression group vs control, *P* < .001) and patients with low NKG7 expression (12.37% ± 1.08% vs 7.00% ± 1.13%, high-expression group vs low-expression group, *P* = .002). No difference was observed between patients with low NKG7 expression and controls (Figure 2G, H). The MFI of CD107a was elevated in patients with high NKG7 expression, compared with controls (4551 ± 382.4 vs 3014 ± 268.8, high-expression group vs control, *P* = .002) and patients with low NKG7 expression (4551 ± 382.4 vs 3132 ± 401.2, high-expression group vs low-expression group, *P* = .02). There was no difference between patients with low NKG7 expression and controls (Figure 2I).

Platelet apoptosis in patients with high NKG7 expression was higher than both controls (9.43% ± 0.80% vs 2.66% ± 0.45%, high-expression group vs control, *P* < .001) and patients with low NKG7 expression (9.43% ± 0.80% vs 4.22% ± 0.85%, high-expression group vs low-expression group, *P* < .001). There was no difference between patients with low NKG7 expression and controls (Figure 2J, K) (NKG7 high-expression group: *n* = 27, NKG7 low-expression group: *n* = 16, control: *n* = 23).

### 3.4 Platelet counts in peripheral blood were significantly decreased in patients with high NKG7 expression

**Figure 3:**
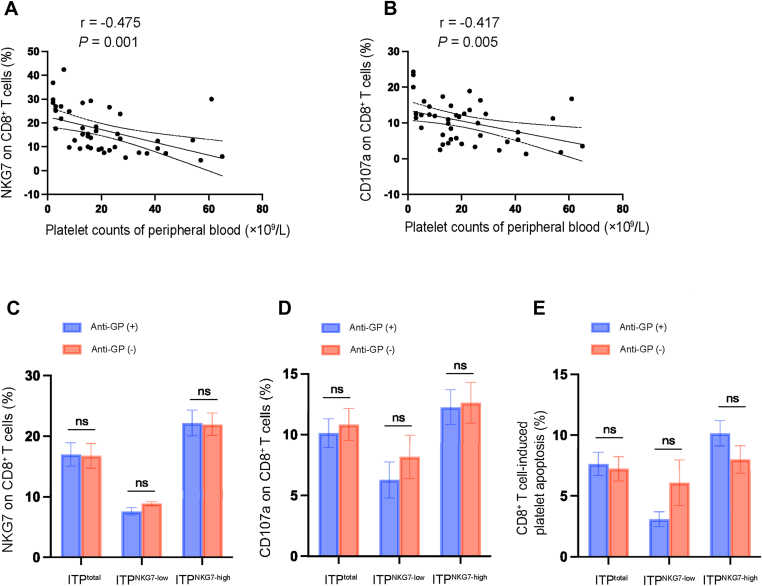
Natural killer cell granule protein 7 (NKG7) and CD107a expression are both negatively correlated with peripheral blood platelet counts in immune thrombocytopenia (ITP). (A) There was a negative correlation between NKG7 and peripheral platelet counts in patients with ITP (*r* = −0.475, *P* = .001); *n* = 43. (B) There was a negative correlation between CD107a expression and peripheral platelet counts in patients with ITP (*r* = −0.417, *P* = .005); n = 43. (C) NKG7 expression was not different between patients with and without antiplatelet glycoprotein antibodies in total patients (*P* = .94), NKG7 low-expression patients (*P* = .1), and NKG7 high-expression patients (*P* = .95). (D) CD107a expression was not different between patients with and without platelet antibodies in total patients (*P* = .71), NKG7 low-expression patients (*P* = .44), and NKG7 high-expression patients (*P* = .88). (E) CD8^+^ T cell-mediated platelet apoptosis was not different between patients with and without platelet antibodies in total patients (*P* = .80), NKG7 low-expression patients (*P* = 0.12), and NKG7 high-expression patients (*P* = 0.21). ns, not significant. Anti-GP, antiplatelet glycoprotein antibodies (anti–GP Ib/Ⅸ and anti–GP IIb/IIIa); Anti-GP (+), patients with antiplatelet glycoprotein antibodies. Anti-GP (−), patients without antiplatelet glycoprotein antibodies. Total ITP group: *n* = 43, NKG7 high-expression group: *n* = 27, NKG7 low-expression group: *n* = 16.

We collected demographic characteristics and medical data of patients and controls. As shown in Tables 1 and 2, there was no significance in age and sex. The platelet counts were much lower in NKG7 high-expression group than those in low-expression group (median 13; IQR, 5, 23 vs 24; IQR, 17, 40, high-expression group vs low-expression group, *P* = .004; Table 2). We compared the correlation between NKG7 expression and platelet counts, as well as the correlation between CD107a expression and platelet counts, in patients with ITP. NKG7 expression was negatively correlated with platelet counts (*r* = −0.475, *P* = .001; Figure 3A). Similarly, there was also a negative correlation between CD107a expression and platelet counts (*r* = −0.417, *P* = .005; Figure 3B) (NKG7 high-expression group: *n* = 27, NKG7 low-expression group: *n* = 16, control: *n* = 23).

**Table 1:** Cohort characteristics.

Characteristic	ITP (*n* = 43)	Controls (*n* = 23)
Age (y), median (IQR)	47 (33, 63)	40 (33, 51)
Female, *n* (%)	23 (53.5%)	12 (52.2%)
Platelet count (×10^9^/L), median (IQR)	16 (8, 27)	251 (208, 284)

ITP, immune thrombocytopenia.

**Table 2:** Demographic characteristics and clinical data of patients with ITP with low and high NKG7 expression.

Characteristic	NKG7 ^low-expression^ (*n* = 16)	NKG7 ^high-expression^ (*n* = 27)	*P*
Age (y)	55 (32, 66)	38 (33, 60)	.31^a^
Female sex, % (*n*)	50 (8)	56 (15)	.72^b^
Platelet counts, (× 10^9^/L)	24 (17, 40)	13 (5, 23)	.004^a^
Bleeding score	1 (0, 3)	2 (1, 3)	.38^a^
Lymphocyte counts, (×10^9^/L)	1.97 (0.94, 2.79)	1.69 (1.16, 2.07)	.34^a^
MAIPA, % (*n*)			
Anti–GP Ib/Ⅸ positive	13 (2)	19 (5)	.93^b^
Anti–GP IIb/IIIa positive	31 (5)	30 (8)	.91^b^
Double positive	19 (3)	19 (5)	.99^b^
Negative	38 (6)	33 (9)	.78^b^

Values are median (IQR) unless otherwise indicated.

GP, glycoprotein; ITP, immune thrombocytopenia; MAIPA, monoclonal antibody-specific immobilization of platelet antigen; NKG7, natural killer cell granule protein 7.

a Mann–Whitney U-test.

b Chi-squared test.

The majority of patients with ITP presented with single or multiple bleeding symptoms, including skin, mucosae, and organ bleeding. Some patients had no bleeding symptoms, and thrombocytopenia was found during routine physical examination. Bleeding scores were calculated at the time of admission, and more information is shown in Table 3 (*n* = 43).

**Table 3:** Summary of bleeding scores for 43 patients with immune thrombocytopenia.

Bleeding score	% (*n*)
0	18 (8)
1	28 (12)
2	18 (8)
3	14 (6)
4	5 (2)
5	5 (2)
6	5 (2)
7	2 (1)
8	5 (2)

Of the 43 patients with ITP, antiplatelet glycoprotein antibodies (antibodies against platelet glycoprotein Ib/Ⅸ, Ⅱb/Ⅲa, or both) were detected in 28 patients (65.12%). In terms of each group, among the 16 patients with low NKG7 expression, antiplatelet glycoprotein antibodies were detected in 10 patients. In addition, among the 27 patients with high NKG7 expression, antiplatelet glycoprotein antibodies were detected in 18 patients. The distribution and proportions of antibodies were presented in Table 2. We found that there was no difference in age, sex, platelet counts, or bleeding scores between patients with and without platelet antibodies among the total patients, patients with low NKG7 expression, and those with high expression, as shown in Table 4. Additionally, we examined the effects of antiplatelet glycoprotein antibodies on NKG7 expression, CD107a expression, and CD8^+^ T cell-mediated platelet apoptosis in these 3 groups. No significant difference was found in the expression levels of the above indicators between patients with and without platelet antibodies (Figure 3C–E). In other words, antiplatelet glycoprotein antibodies did not affect the expression of NKG7 and CD107a or the proportion of CD8^+^ T cell-mediated platelet apoptosis (total ITP group: *N* = 43, NKG7 high-expression group: *n* = 27, NKG7 low-expression group: *n* = 16).

**Table 4:** Demographic characteristics and clinical data of patients with and without antiplatelet glycoprotein antibodies.

Characteristic	Total patients (*N* = 43)			NKG7 ^low-expression^ (*n* = 16)			NKG7 ^high-expression^ (*n* = 27)		
	Anti-GP (+) (*n* = 28)	Anti-GP (−) (*n* = 15)	*P*	Anti-GP (+) (*n* = 10)	Anti-GP (−) (*n* = 6)	*P*	Anti-GP (+) (*n* = 18)	Anti-GP (−) (*n* = 9)	*P*
Age (y)	41 (29, 63)	52 (36, 65)	.23^a^	61 (30, 67)	49 (36, 60)	.49^a^	37 (24, 58)	54 (36, 70)	.07^a^
Female sex, % (*n*)	54 (15)	53 (8)	.99^b^	50 (5)	50 (3)	.70^b^	56 (10)	56 (5)	0.66^b^
Platelet counts, (× 10^9^/L)	16 (7, 35)	18 (13, 23)	.93^a^	27 (15, 47)	22 (18, 36)	.64^a^	13 (5, 26)	15 (6, 21)	0.93^a^
Bleeding score	2 (1, 4)	1 (1, 2)	.46^a^	1 (0, 4)	2 (1, 4)	.77^a^	2 (1, 5)	1 (1, 2)	0.22^a^

Values are median (IQR) unless otherwise specified.

Anti-GP, antiplatelet glycoprotein antibodies (anti-GP Ib/Ⅸ and anti-GP IIb/IIIa). Anti-GP (+), patients with antiplatelet glycoprotein antibodies. Anti-GP (−), patients without antiplatelet glycoprotein antibodies.

a Mann–Whitney U-test.

b Chi-squared test.

### 3.5 NKG7 knockdown attenuated CD8^+^ T cell-mediated cytotoxicity

**Figure 4:**
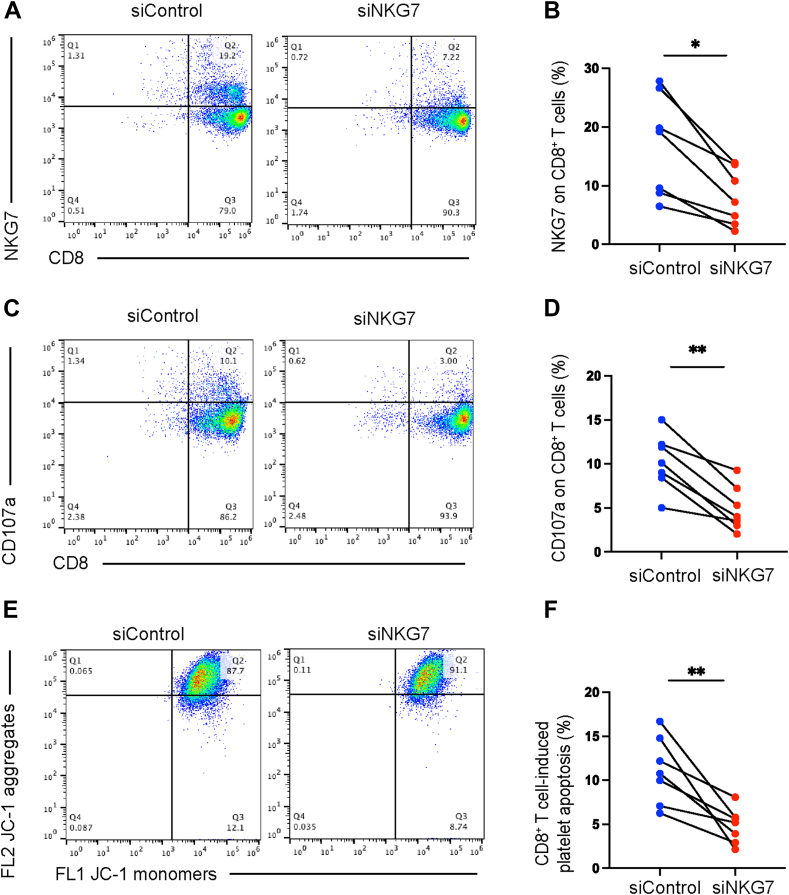
Natural killer cell granule protein 7 (*NKG7*) knockdown attenuates CD8^+^ T cell-mediated cytotoxicity. (A) Flow cytometric analysis of NKG7 expression on CD8^+^ T cells without or with small interfering NKG7 (siNKG7). (B) Expression of NKG7 was significantly decreased in the siNKG7 group compared with the control group (*P* = .04). (C) Flow cytometric analysis of CD107a expression between control group and siNKG7 group. (D) Compared with control group, the expression of CD107a was obviously reduced in siNKG7 group (*P* = 0.005). (E) Flow cytometric figures of CD8^+^ T cell-induced platelet ΔΨ_m_ depolarization between control group and siNKG7 group. JC-1 aggregates reflect potential-dependent accumulation in the mitochondria, whereas JC-1 monomers represent the monomeric form of JC-1 after mitochondrial ΔΨ_m_ depolarization. Platelet apoptosis can be assessed by measuring the percentage of JC-1 monomers only. (F) Platelet apoptosis in siNKG7 group was significantly lower than in the control group (*P* = .002). *∗P <* 0.05; *∗∗P <* 0.01. siNKG7, siRNA-mediated knockdown of NKG7; siControl, siRNA-mediated knockdown of negative control. *n* = 7.

**Supplementary FigS2:**
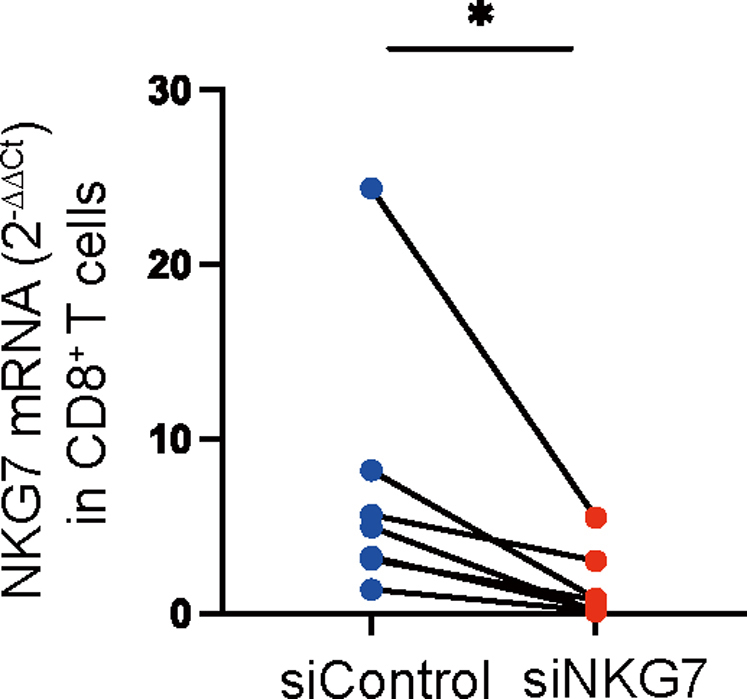


To further investigate the role of NKG7 in CD8^+^ T cells in ITP, we knocked down NKG7 in CD8^+^ T cells by siRNA. *NKG7* mRNA expression was decreased significantly after NKG7 knockdown (4.97; IQR, 3.09, 8.22 vs 0.79; IQR, 0.17, 3.04, siControl vs siNKG7, *P* = 0.01; Supplementary Figure 2]. We found that compared with siControl group, CD8^+^ T cells in siNKG7 group exhibited decreased expression of NKG7 (16.90% ± 3.29% vs 8.01% ± 1.81%, siControl vs siNKG7, *P* = .04; Figure 4A, B) and CD107a (10.23% ± 1.21% vs 4.91% ± 0.96%, siControl vs siNKG7, *P* = .005; Figure 4C, D), along with decreased platelet apoptosis (11.10% ± 1.44% vs 4.80% ± 0.76%, siControl vs siNKG7, *P* = .002; Figure 4E, F). (*n* = 7).

### 3.6 NKG7 regulated CD8^+^ T cell-mediated cytotoxicity by the ERK1/2 pathway in ITP

**Figure 5:**
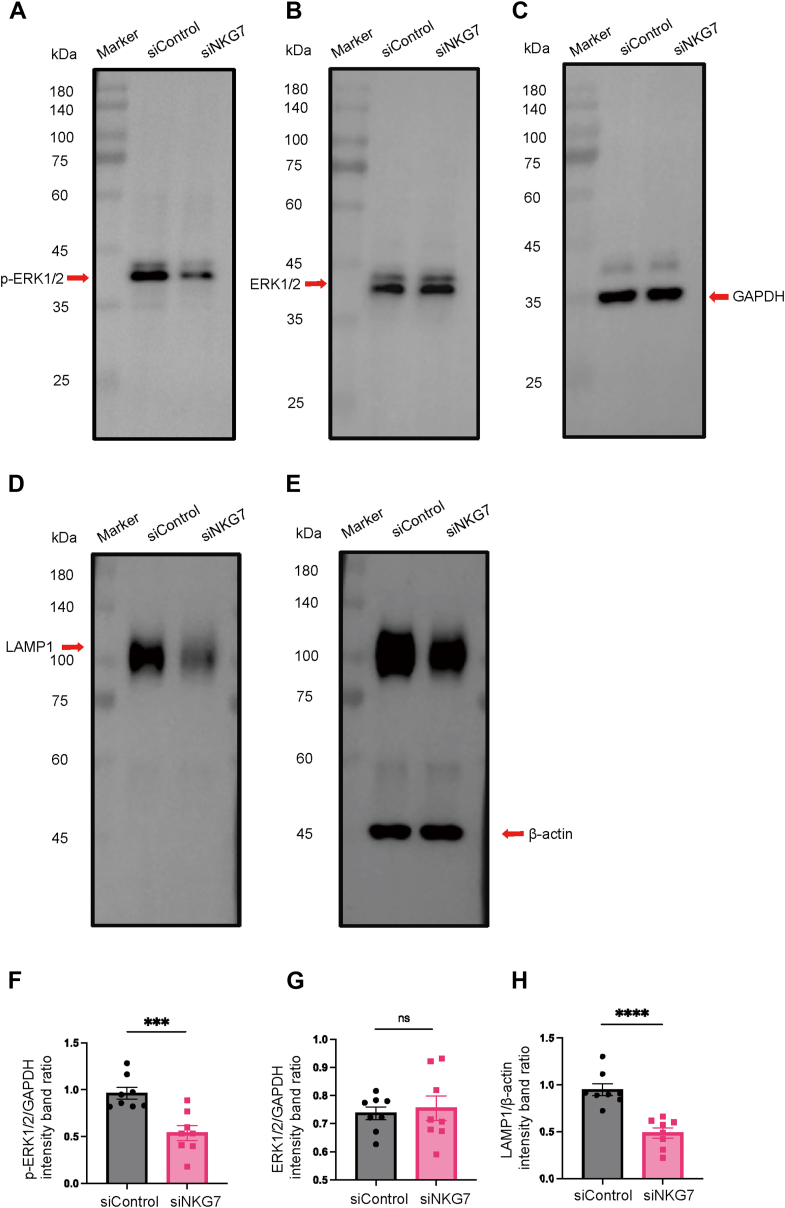
Natural killer cell granule protein 7 (NKG7) affects CD8^+^ T cell-mediated cytotoxicity via the extracellular signal-regulated kinases 1 and 2 (ERK1/2) pathway in immune thrombocytopenia (ITP). Western blot analysis of (A) phosphorylated (p)-ERK1/2, (B) total ERK1/2, (C) glyceraldehyde-3-phosphate dehydrogenase (GAPDH), (D) lysosome-associated membrane glycoprotein 1 (LAMP1), and (E) β-actin. GAPDH and β-actin were used as endogenous controls. (F) The relative expression of p-ERK was significantly decreased in the siRNA-mediated knockdown of NKG7 (siNKG7) group compared with the control group (*P <* .001). (G) There was no difference in the relative expression of total ERK between the siNKG7 group and the control group (*P* = .72). (H) The relative expression of LAMP1 was significantly reduced in the siNKG7 group compared with the control group (*P <* .001). ∗∗∗*P* < .001; ∗∗∗∗*P* < .0001; ns, not significant. *n* = 8.

ERK1/2 signaling plays an important role in cell activation, proliferation, differentiation, and survival [42,43]. In CD8^+^ T cells and NK cells, the ERK1/2 signaling pathway is involved in regulating the expression of lytic granules toward target cells [44,45]. To investigate the ERK pathway after the knockdown of NKG7, we analyzed the protein expression in CD8^+^ T cells by western blot analysis (Figure 5A–E). We found that the level of ERK1/2 phosphorylation in CD8^+^ T cells was attenuated significantly by NKG7 knockdown (0.96 ± 0.06% vs 0.54% ± 0.08%, siControl vs siNKG7, *P* < .001; Figure 5F). However, the level of total ERK1/2 did not change regardless of whether NKG7 was knocked down (0.74 ± 0.02% vs 0.76% ± 0.04%, siControl vs siNKG7, *P* = .72; Figure 5G). In addition, the level of LAMP1 was also decreased significantly in CD8^+^ T cells after NKG7 knockdown (0.95 ± 0.06% vs 0.49% ± 0.06%, siControl vs siNKG7, *P* < .001; Figure 5H). (*n* = 8).

### 3.7 NKG7 expression in NK cells was not higher in patients with ITP than in controls

**Figure 6:**
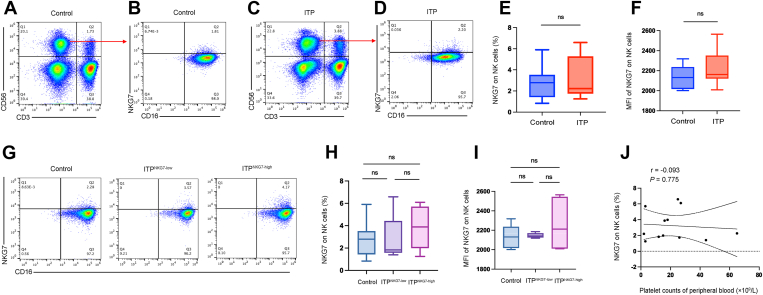
Natural killer cell granule protein 7 (NKG7) expression in natural killer (NK) cells is not higher in patients with immune thrombocytopenia (ITP) than in controls. (A, B) Flow cytometric analysis of NKG7 expression in NK cells of healthy controls. (C, D) Flow cytometric analysis of NKG7 expression in NK cells of patients with ITP. (E) There was no difference in NKG7 proportion in NK cells between patients with ITP and controls (*P* = .50). (F) The mean fluorescence intensity (MFI) of NKG7 expression did not differ between patients with ITP and controls (*P* = .22). (G) Flow cytometric analysis of NKG7 in NK cells in controls, NKG7 low-expression patients, and NKG7 high-expression patients. (H) The expression of NKG7 did not differ between NKG7 high-expression patients and controls (*P* = .31), as well as between NKG7 low-expression patients and controls (*P* = .97). No significant difference was found between NKG7 high-expression patients and low-expression patients (*P* = 0.50). (I) NKG7 MFI did not differ between NKG7 high-expression patients and controls (*P* = .13), as well as between NKG7 high-expression patients and low-expression patients (*P* = 0.26). There was no difference between NKG7 low-expression patients and controls (*P* = 0.83). (J) There was no correlation between NKG7 expression in NK cells and peripheral platelet counts (*r* = −0.093, *P* = .78). ns, not significant. Control: *n* = 10, NKG7 low-expression group: *n* = 5, NKG7 high-expression group: *n* = 7.

We evaluated NKG7 expression on NK cells in patients with ITP and controls by flow cytometry. There was no difference between patients and controls (3.24% ± 0.56% vs 2.73% ± 0.48%, patients vs controls, *P* = .50; Figure 6A–E). The MFI of NKG7 was not different between patients with ITP and controls (2220 ± 53.47 vs 2135 ± 36.39, patients vs controls, *P* = .22; Figure 6F). We further analyzed NKG7 expression in controls, patients with low NKG7 expression, and patients with high NKG7 expression. We found that there was no difference between patients with high NKG7 expression and controls (3.59% ± 0.71% vs 2.73% ± 0.48%, high-expression group vs control, *P* = .31), as well as between patients with low NKG7 expression and controls (2.76% ± 0.97% vs 2.73% ± 0.48%, low-expression group vs control, *P* = .97). No significant difference was found between patients with high NKG7 expression and those with low expression (3.59% ± 0.71% vs 2.76% ± 0.97%, high-expression group vs low-expression group, *P* = .50; Figure 6G, H). Similarly, there was no significant difference in the MFI of NKG7 expression among the 3 groups. We also tested the correlation of NKG7 expression on NK cells with platelet counts in patients with ITP. There was no correlation between these 2 indicators (*r* = −0.093, *P* = .78; Figure 6J). In addition, we also found that the expression of CD107a on NK cells and NK cell-mediated platelet apoptosis were very low (Supplementary Figure 3A–D) (Control: *n* = 10, NKG7 low-expression group: *n* = 5, NKG7 high-expression group: *n* = 7).

## 4 Discussion

Previous studies found that NKG7 was associated with cell cytotoxicity [26,46], particularly in the setting of inflammation and autoimmune diseases, as well as in antitumor immunotherapy [30,31,47]. Our results show that NKG7 has a close correlation with CD8^+^ T cell-mediated cytotoxicity in ITP, including the release of lytic granules and platelet apoptosis. In the present study, both protein and gene expression levels of NKG7 in CD8^+^ T cells from patients with ITP were significantly higher than those in healthy controls. Several studies have demonstrated that the expression of NKG7 was increased in CD8^+^ T cells in patients with type 1 diabetes mellitus and those with progressive with IgA nephropathy [47,48]. NKG7 expression in CD8^+^ T cells was also elevated in some cancer patients who showed durable response to immunotherapy [33]. However, NKG7 expression was decreased in patients with ankylosing spondylitis [49]. These results indicate that the effect of NKG7 is complex and NKG7 might show different effects in different diseases.

The cytotoxicity of CD8^+^ T cells is mainly dependent on degranulation. CD107a (LAMP-1) is considered as a marker of degranulation in cytotoxicity [24]. Previous reports have suggested that the appearance of CD107a on antigen-specific CD8^+^ T cells is associated with loss of intracellular perforin and directly correlated with cytotoxic activity [23]. NKG7 expression is confined to CD8^+^ T cells and NK cells, with relatively minimal expression by CD4^+^ T cells [28,30,35,50]. Moreover, Huang et al. [51] confirmed that NKG7 was highly expressed in the early stages of regulatory T cell differentiation. NKG7 colocalizes with CD107a on the luminal side of cytotoxic granules and translocates from granules to the plasma membrane during cytotoxic cell degranulation [26,30]. NKG7 deficiency results in reduced CD107a production and impairs tumor cell killing [30,31,35]. In the last 2 decades, many studies have discovered CD8^+^ T cell-mediated lysis of platelets in patients with ITP [[12], [13], [14],^19^]. In our study, we used both the expression of CD107a and the percentage of CD8^+^ T cell-induced platelet apoptosis as indicators of CD8^+^ T cell-mediated cytotoxicity. As expected, NKG7 expression was positively correlated with CD107a and platelet apoptosis. This result suggests that NKG7 may play an important role in CD8^+^ T cell-mediated cytotoxicity in ITP.

As shown in Figure 1B, not all patients had higher expression of NKG7 than healthy controls. To explore the profile of NKG7 in patients with ITP, we divided patients into 2 groups: NKG7 high-expression group and NKG7 low-expression group. We found that the NKG7 high-expression group had significantly higher levels of CD8^+^ T cell-mediated cytotoxicity than controls; however, there was no difference between the NKG7 low-expression group and control group. This result confirms that ITP is a heterogeneous disease [8,52,53]. Various mechanisms can participate in the pathogenesis of thrombocytopenia, eg, platelet autoantibodies, abnormal T cell immunity, disturbed cytokines, and impaired megakaryocyte maturation [6,[54], [55], [56], [57]]. Recent studies have shown that exosomes of patients with ITP can induce complement system dysfunction, promote inflammatory reactions, and attenuate regulatory T cell-mediated immune suppression [50,51]. These abnormal conditions can all exacerbate platelet destruction or reduce platelet production. For patients with low NKG7 expression, CD8^+^ T cell-mediated cytotoxicity could not be regarded as the main cause of thrombocytopenia. Compared to patients with low NKG7 expression, patients with high NKG7 expression had much lower platelet counts in peripheral blood. We analyzed the relationship between NKG7 expression and platelet counts and discovered a negative correlation. Due to the lower platelet counts and higher disease severity, the NKG7 high-expression group may require more proactive intervention. Therefore, NKG7 can be used as an important protein for predicting disease severity. Zhao et al. [58] reported that CD8^+^ T cell-mediated cytotoxicity was increased in patients with ITP who did not have antiplatelet glycoprotein antibodies. In our study, we compared the levels of NKG7 and CD107a expression and CD8^+^ T cell-mediated platelet apoptosis in patients with or without antiplatelet glycoprotein antibodies. However, we did not find any significant difference.

To further investigate the role of NKG7 in CD8^+^ T cells, we performed knockdown of NKG7 using siRNA in CD8^+^ T cells from patients from ITP. CD8^+^ T cells with NKG7 knockdown exhibited decreased CD107a expression and CD8^+^ T cell-induced platelet apoptosis. ERK1/2 is a pivotal serine/threonine kinase belonging to the mitogen-activated protein kinase family. The ERK pathway widely exists in NK cells, T/B lymphocytes, and megakaryocytes and plays an important role in cell proliferation, differentiation, and survival [43,44,[59], [60], [61]]. Furthermore, ERK signaling is necessary for regulating the expression of perforin and granzyme B in CD8^+^ T cells [45]. In the present study, we found that after NKG7 knockdown, the phosphorylation of ERK1/2 and LAMP-1 was significantly attenuated; however, total ERK1/2 was not affected. This result shows that in patients with ITP, NKG7 might affect CD8^+^ T cell-mediated cytotoxicity via the ERK1/2 signaling pathway.

We detected the expression of NKG7 in NK cells from patients with ITP and healthy individuals. NKG7 expression was not increased in patients compared with controls. The level of NKG7 was not correlated with platelet counts. Additionally, CD107a expression in NK cells and NK cell-mediated platelet apoptosis were very low in ITP. We consider there are 2 possible reasons. First, the low proportion of NK cells in PBMCs may result in their relatively weak cytotoxic activity [[62], [63], [64], [65]]. Second, platelets predominantly express MHC-I and T cell costimulatory molecules, while NK cells primarily exert their effects through antibody-dependent cell-mediated cytotoxicity, leading to reduced NK cell-mediated cytotoxicity against platelets [[66], [67], [68]].

Nonimmune thrombocytopenia, primarily caused by solid tumors, chemotherapy, and poor platelet engraftment, involves complex CD8^+^ T cell dynamics [69]. Tumor surface antibodies activate antigen-specific CD8^+^ T cells, the infiltration and activity of which vary greatly in different types of tumors [70,71]. Chemotherapy alters the tumor microenvironment, inducing CD8^+^ T cell proliferation and migration toward myeloid cell-rich areas, where myeloid cells exhaust these T cells and suppress cytotoxicity [72]. Prolonged radiotherapy/chemotherapy in patients with hematologic malignancies damages bone marrow stroma, contributing to nonimmune platelet engraftment failure [73,74]. The function and activity of CD8^+^ T cells in the complex transplantation environment may be different. Therefore, in nonimmune thrombocytopenia, how CD8^+^ T cells function and how NKG7 influences the function of CD8^+^ T cells are interesting questions worthy of further exploration.

Our study has some limitations. Patients with ITP were recruited from a single center, and the sample size was not large enough; hence, these data might not be representative of the general population. We did not investigate the role of NKG7 in CD8^+^ T cells in nonimmune thrombocytopenia. This limits the research scope of our study, making it lack universal applicability. More detailed and thorough studies are required in the future.

## 5 Conclusions

In conclusion, our results show that NKG7 plays an important role in CD8^+^ T cell-mediated cytotoxicity in ITP, including enhancing the potential of degranulation and lysing platelets. This provides a possibility for the future application of targeting NKG7 for the treatment of ITP.

## Figures and Tables

**Supplementary FigS3:**